# Supplementary material for: The interaction between cancer and COVID-19: Risk factors and targeted interventions
Source: PLoS One. 2025 Jun 3;20(6):e0319970. doi: 10.1371/journal.pone.0319970 (PMC12132958; doi:10.1371/journal.pone.0319970)
Supplement: S1 Table — (DOCX) [file pone.0319970.s001.docx]

Table S1. Supplementary table: Interaction of factors associated with mechanical ventilation, ICU and death from COVID-19.

|  | Yes (%) | No (%) | OR | IC95% | *P* | Interaction measures |  |
| --- | --- | --- | --- | --- | --- | --- | --- |
| **MECHANICAL VENTILATION** | | | | | | |  |
| **Comorbidity / tumor type**^1^ |  |  |  |  |  |  |  |
| No / solid | 44 (28) | 381 (44.4) |  | 1.00 |  |  |  |
| No / hematologic | 10 (6.4) | 26 (3) | 3.39 | (1.53 - 7.51) | <0.01 | RERI = -1.18 (-4.27 - 1.91) |  |
| Yes / solid | 86 (54.8) | 402 (46.8) | 1.79 | (1.21 - 2.65) | <0.01 | AP = -0.39 (-1.54 - 0.75) |  |
| Yes / hematologic | 17 (10.8) | 50 (5.8) | 3.00 | (1.59 - 5.66) | <0.01 | SI = 0.63 (0.20 - 1.98) |  |
| **ICU** | | | | | | |  |
| **Comorbidity / tumor type**^1^ |  |  |  |  |  |  |  |
| No / solid | 84 (31.6) | 356 (44.7) |  | 1.00 |  |  |  |
| No / hematologic | 17 (6.4) | 25 (3.1) | 2.86 | (1.47 - 5.54) | <0.01 | RERI = -0.40 (-2.71 - 1.90) |  |
| Yes / solid | 136 (51.1) | 374 (46.9) | 1.51 | (1.11 - 2.07) | <0.01 | AP = -0.14 (-0.95-0.68) |  |
| Yes / hematologic | 29 (10.9) | 42 (5.3) | 2.97 | (1.75 - 5.05) | <0.01 | SI = 0.83 (0.29 - 2.35) |  |
| **DEATH FROM COVID-19** | | | | | | |  |
| **Age group / Multimorbidity**^2^ |  |  |  |  |  |  |  |
| <60 years / No | 192 (22.4) | 155 (32.3) |  | 1.00 |  |  |  |
| <60 years / yes | 108 (12.6) | 58 (12.1) | 1.43 | (0.92 - 2.21) | 0.11 | RERI = 0.04 (-0.73 - 0.82) |  |
| >60 years / no | 291 (34) | 159 (33.1) | 1.32 | (0.93 - 1.86) | 0.12 | AP = 0.02 (-0.41 - 0.46) |  |
| >60 years / yes | 265 (31) | 108 (22.5) | 1.78 | (1.24 - 2.56) | <0.01 | SI = 1.06 (0.37 - 2.99) |  |
| **Age group / Tumor type**^3^ |  |  |  |  |  |  |  |
| <60 years / hematologic | 34 (4) | 36 (7.5) |  | 1.00 |  |  |  |
| <60 years / solid | 266 (31.1) | 177 (36.9) | 1.67 | (0.89 - 3.11) | 0.1 | RERI = 0.19 (-0.86 - 1.23) |  |
| >60 years / hematologic | 56 (6.5) | 28 (5.8) | 1.36 | (0.61 - 3.05) | 0.45 | AP = 0.08 (-0.40 - 0.57) |  |
| >60 years / solid | 500 (58.4) | 239 (49.8) | 2.22 | (1.21 - 4.07) | 0.01 | SI = 1.18 (0.40 - 3.52) |  |

Supplementary Table (continued)

| **DEATH FROM COVID-19 (continued)** | | | | | | |
| --- | --- | --- | --- | --- | --- | --- |
| **Age group / vaccination period**^4^ |  |  |  |  |  |  |
| <60 years / after vaccination | 84 (9.8) | 90 (18.8) |  | 1.00 |  |  |
| <60 years / before vaccination | 216 (25.2) | 123 (25.6) | 2.09 | (1.35 - 3.22) | <0.01 | RERI = 0.23 (-0.70 - 1.15) |
| >60 years / after vaccination | 170 (19.9) | 136 (28.3) | 1.39 | (0.91 - 2.13) | 0.13 | AP = 0.08 (-0.26 - 0.42) |
| >60 years / before vaccination | 386 (45.1) | 131 (27.3) | 2.71 | (1.80 - 4.08) | <0.01 | AP = 0.08 (-0.26 - 0.42) |
| **Age group / mechanical ventilation**^5^ |  |  |  |  |  |  |
| <60 years / no | 180 (28.6) | 148 (38.3) |  | 1.00 |  |  |
| <60 years / yes | 38 (6) | 12 (3.1) | 0.96 | (0.64 - 1.46) | 0.87 | RERI = -0.36 (-1.01 - 0.29) |
| >60 years / no | 329 (52.2) | 202 (52.3) | 1.50 | (1.06 - 2.13) | 0.02 | AP = -0.33 (-0.92 - 0.26) |
| >60 years / yes | 83 (13.2) | 24 (6.2) | 1.10 | (0.74 - 1.64) | 0.62 | SI = 0.22 (0.01 - 6.88) |
| **Multimorbidity / tumor type**^6^ |  |  |  |  |  |  |
| No / hematologic | 42 (4.9) | 48 (10.0) |  | 1.00 |  |  |
| No / solid | 441 (51.5) | 266 (55.4) | 2.76 | (1.52 - 5.01) | <0.01 | RERI = -2.04 (-5.04 - 0.97) |
| Yes / hematologic | 48 (5.6) | 16 (3.3) | 3.67 | (1.58 - 8.54) | <0.01 | AP = -0.60 (-1.40 - 0.19) |
| Yes / solid | 325 (38) | 150 (31.3) | 3.39 | (1.84 - 6.24) | <0.01 | **SI = 0.54 (0.29 - 0.99)** |
| **Multimorbidity / vaccination period**^7^ |  |  |  |  |  |  |
| No / after vaccination | 147 (17.2) | 144 (30) |  | 1.00 |  |  |
| No / before vaccination | 336 (39.3) | 170 (35.4) | 1.76 | (1.25 - 2.49) | <0.01 | RERI = 0.80 (-0.11 - 1.72) |
| Yes / after vaccination | 107 (12.5) | 82 (17.1) | 1.17 | (0.77 - 1.77) | 0.45 | **AP = 0.29 (0.01 - 0.58)** |
| Yes / before vaccination | 266 (31.1) | 84 (17.5) | 2.74 | (1.88 - 4.00) | <0.01 | SI = 1.86 (0.82 - 4.25) |
| **Multimorbidity / mechanical ventilation**^8^ |  |  |  |  |  |  |
| No / no | 280 (44.4) | 215 (55.7) |  | 1.00 |  |  |
| No / yes | 55 (8.7) | 25 (6.5) | 1.89 | (1.09 - 3.07) | 0.02 | RERI = 2.47 (-0.66 - 5.60) |
| Yes / no | 229 (36.3) | 135 (35) | 1.28 | (0.96 - 1.70) | 0.09 | **AP = 0.54 (0.18 - 0.98)** |
| Yes / yes | 66 (10.5) | 11 (2.8) | 4.58 | (2.33 - 9.02) | <0.01 | SI = 3.22 (0.97 - 10.75) |

Supplementary Table (continued)

| **DEATH FROM COVID-19 (continued)** | | | | | | |
| --- | --- | --- | --- | --- | --- | --- |
| **Tumor type / vaccination period**^9^ |  |  |  |  |  |  |
| Hematological / after vaccination | 30 (3.5) | 30 (6.3) |  | 1.00 |  |  |
| Hematological / before vaccination | 60 (7.0) | 34 (7.1) | 1.96 | (0.87 - 4.40) | 0.10 | RERI = 0.68 (-0.59 - 1.95) |
| Solid / after vaccination | 224 (26.2) | 196 (40.8) | 1.66 | (0.87 - 3.19) | 0.12 | AP = 0.21 (-0.20 - 0.61) |
| Solid / before vaccination | 542 (63.3) | 220 (45.8) | 3.30 | (1.74 - 6.27) | <0.01 | SI = 1.42 (0.59 - 3.41) |
| **Tumor type / mechanical ventilation**^10^ |  |  |  |  |  |  |
| Hematological / no | 32 (5.1) | 44 (11.4) |  | 1.00 |  |  |
| Hematological / yes | 22 (3.5) | 5 (1.3) | 5.96 | (2.01 - 17.64) | <0.01 | RERI = -2.88 (-9.24 - 3.47) |
| Solid / no | 477 (75.7) | 306 (79.3) | 2.08 | (1.28 - 3.39) | <0.01 | AP = -0.69 (-2.27 - 0.88) |
| Solid / yes | 99 (15.7) | 31 (8.0) | 4.15 | (2.23 - 7.72) | <0.01 | SI = 0.52 (0.17 - 1.58) |
| **Vaccination period / mechanical ventilation**^11^ |  |  |  |  |  |  |
| after vaccination / no | 162 (25.7) | 169 (43.8) |  | 1.00 |  |  |
| after vaccination / yes | 40 (6.3) | 17 (4.4) | 2.61 | (1.41 - 4.82) | <0.01 | RERI = 0.87 (-1.87 - 3.61) |
| before vaccination / no | 347 (55.1) | 181 (46.9) | 2.02 | (1.52 - 2.68) | <0.01 | AP = 0.19 (-0.34 - 0.73) |
| before vaccination / yes | 81 (12.9) | 19 (4.9) | 4.50 | (2.59 - 7.80) | <0.01 | SI = 1.33 (0.55 - 3.20) |

1 - Adjusted for: age group and vaccination period.

2 - Adjusted for: tumor type, vaccination period and mechanical ventilation.

3 - Adjusted for: vaccination period and mechanical ventilation.

4 - Adjusted for: tumor type and mechanical ventilation.

5 - Adjusted for: tumor type and vaccination period.

6 - Adjusted for: age group, vaccination period and mechanical ventilation.

7 - Adjusted for: age group, tumor type and mechanical ventilation.

8 - Adjusted for: age group, tumor type and vaccination period.

9 - Adjusted for: age group, multimorbidity and mechanical ventilation.

10 - Adjusted for: age group, multimorbidity and vaccination period.

11 - Adjusted for: age group, tumor type and multimorbidity.
